# HIV multi-drug resistance at first-line antiretroviral failure and subsequent virological response in Asia

**DOI:** 10.7448/IAS.17.1.19053

**Published:** 2014-08-19

**Authors:** Awachana Jiamsakul, Somnuek Sungkanuparph, Matthew Law, Rami Kantor, Jutarat Praparattanapan, Patrick CK Li, Praphan Phanuphak, Tuti Merati, Winai Ratanasuwan, Christopher KC Lee, Rossana Ditangco, Mahiran Mustafa, Thida Singtoroj, Sasisopin Kiertiburanakul, on behalf of the TREAT Asia Studies to Evaluate Resistance – Monitoring Study (TASER-M)

**Affiliations:** 1 The Kirby Institute, UNSW Australia, Sydney, Australia; 2 Faculty of Medicine Ramathibodi Hospital, Mahidol University, Bangkok, Thailand; 3 Division of Infectious Diseases, Brown University Alpert Medical School, Rhode Island, USA; 4 Research Institute for Health Sciences, Chiang Mai University, Chiang Mai, Thailand; 5 Department of Medicine, Queen Elizabeth Hospital, Hong Kong, China; 6 Faculty of Medicine, Chulalongkorn University, Bangkok, Thailand; 7 HIV-NAT/Thai Red Cross AIDS Research Centre, Bangkok, Thailand; 8 Udayana University, Sanglah Hospital, Bali, Indonesia; 9 Faculty of Medicine Siriraj Hospital, Mahidol University, Bangkok, Thailand; 10 Hospital Sungai Buloh, Sungai Buloh, Malaysia; 11 Research Institute for Tropical Medicine, Manila, Philippines; 12 Hospital Raja Perempuan Zainab II, Kota Bharu, Malaysia; 13 TREAT Asia, amfAR – The Foundation for AIDS Research, Bangkok, Thailand

**Keywords:** HIV, resource-limited, resistance, mutations, failure

## Abstract

**Introduction:**

First-line antiretroviral therapy (ART) failure often results from the development of resistance-associated mutations (RAMs). Three patterns, including thymidine analogue mutations (TAMs), 69 Insertion (69Ins) and the Q151M complex, are associated with resistance to multiple-nucleoside reverse transcriptase inhibitors (NRTIs) and may compromise treatment options for second-line ART.

**Methods:**

We investigated patterns and factors associated with multi-NRTI RAMs at first-line failure in patients from The TREAT Asia Studies to Evaluate Resistance – Monitoring study (TASER-M), and evaluated their impact on virological responses at 12 months after switching to second-line ART. RAMs were compared with the IAS-USA 2013 mutations list. We defined multi-NRTI RAMs as the presence of either Q151M; 69Ins; ≥2 TAMs; or M184V+≥1 TAM. Virological suppression was defined as viral load (VL) <400 copies/ml at 12 months from switch to second-line. Logistic regression was used to analyze (1) factors associated with multi-NRTI RAMs at first-line failure and (2) factors associated with virological suppression after 12 months on second-line.

**Results:**

A total of 105 patients from 10 sites in Thailand, Hong Kong, Indonesia, Malaysia and Philippines were included. There were 97/105 (92%) patients harbouring ≥1 RAMs at first-line failure, 39/105 with multi-NRTI RAMs: six with Q151M; 24 with ≥2 TAMs; and 32 with M184V+≥1 TAM. Factors associated with multi-NRTI RAMs were CD4 ≤200 cells/µL at genotyping (OR=4.43, 95% CI [1.59–12.37], *p*=0.004) and ART duration >2 years (OR=6.25, 95% CI [2.39–16.36], *p*<0.001). Among 87/105 patients with available VL at 12 months after switch to second-line ART, virological suppression was achieved in 85%. The median genotypic susceptibility score (GSS) for the second-line regimen was 2.00. Patients with ART adherence ≥95% were more likely to be virologically suppressed (OR=9.33, 95% CI (2.43–35.81), *p*=0.001). Measures of patient resistance to second-line ART, including the GSS, were not significantly associated with virological outcome.

**Conclusions:**

Multi-NRTI RAMs at first-line failure were associated with low CD4 level and longer duration of ART. With many patients switching to highly susceptible regimens, good adherence was still crucial in achieving virological response. This emphasizes the importance of continued adherence counselling well into second-line therapy.

## Introduction

In resource-limited settings, first-line antiretroviral therapy (ART) for HIV-positive patients consists of nucleoside and non-nucleoside reverse transcriptase inhibitors (NRTIs and NNRTIs). Protease inhibitors (PIs) are usually the core component of second-line therapy [1]. First-line ART failure is an undesirable treatment outcome often resulting from the development of reverse transcriptase (RT) resistance-associated mutations (RAMs). Much of the data available from resource-limited settings are from Africa, in particular the sub-Saharan Africa. These studies demonstrated that the prevalence of RAMs in patients failing first-line ART ranged from 53 to 84% with 38–64% harbouring dual-class resistance [2–5]. The prevalence of RAMs in individuals experiencing first-line ART failure in Asia has ranged from 87 to 95% with 50–64% harbouring dual-class mutations [6–9].

Three drug resistance patterns including thymidine analogue-associated mutations (TAMs), 69 Insertion (69Ins) and Q151M complex are associated with multi-NRTI drug resistance [10]. TAMs, originally selected by the thymidine analogues zidovudine and stavudine, also confer reduced susceptibility to all approved NRTIs, depending on the number and type of mutations involved. There are two TAM pathways: type I (M41L, L210W and T215Y) and type II (D67N, K70R, T215F and K219Q/E); the former conferring higher levels of resistance and cross-resistance. TAMs are commonly found in low-income countries where thymidine analogue-containing regimens have been prescribed as first-line ART. The 69Ins consists of a substitution at codon 69 and an insertion of two or more amino acids. When it is present with at least one TAM at position 41, 210 or 215, resistance to all approved NRTIs is conferred. Q151M is usually accompanied by the mutations A62V, V75I, F77L and F116Y. The Q151M complex affects all approved NRTIs except for tenofovir. M184V is another important mutation which is also the most commonly occurring NRTI RAM. M184V causes high-level resistance to lamivudine and emtricitabine and low-level resistance to didanosine and abacavir. When present with TAMS, M184V can increase resistance to abacavir [10–12].

The presence of TAMs, 69Ins, Q151M and M184V at first-line ART failure can compromise treatment options for second-line therapy. A case study on one patient from Sierra Leone treated with stavudine, lamivudine and nevirapine reported extensive mutations to all NRTIs and NNRTIs [13]. A study of South African patients found increased risk of drug resistance accumulation with prolonged exposure to the failing ART, which consisted mainly of stavudine-containing regimens [14]. In resource-limited settings, stavudine was commonly used as part of the standard first-line therapy prior to the World Health Organization (WHO) recommendations to phase out the drug due to its toxicities [15]. We investigated the patterns and factors associated with multi-NRTI RAMs, defined as the presence of either Q151M; 69Ins; ≥2 TAMs; or M184V+≥1 TAM, at first-line failure in an Asian HIV cohort where stavudine use was predominant, and evaluated the impact of these RAMS on virological responses at 12 months after switch to second-line ART.

## Methods

### Study population

Patients were selected from The Therapeutics, Research, Education and AIDS Training in Asia (TREAT Asia) Studies to Evaluate Resistance – Monitoring Study (TASER-M). TASER-M began recruitment in 2007 and included 12 clinical sites in Thailand, Hong Kong, Malaysia, Philippines and Indonesia. Patients were either enrolled in TASER-M as treatment naïve initiating first-line ART or treatment-experienced switching to second-line ART due to failure [16]. Patients included in this analysis were either (1) enrolled as treatment-experienced prior to switching to a second-line regimen or (2) enrolled as treatment naïve and failed first-line ART; both groups had an available *pol* genotype (FASTA file) within six months prior to switch to second-line. First-line failures were obtained from the ART stop reasons reported in the database, and included treatment failure, clinical progression or hospitalization.

Data transfers from clinical sites were aggregated at The Kirby Institute, UNSW Australia (The University of New South Wales), Sydney, Australia. Ethics approvals were obtained from UNSW Australia Ethics Committee and institutional review boards at the participating clinical sites and coordinating centre (TREAT Asia/amfAR, Bangkok, Thailand). Written informed consent was obtained from participants prior to enrolment.

### Genotyping

Genotyping was performed at laboratories certified by TREAT Asia Quality Assessment Scheme (TAQAS). TAQAS was developed as a quality assessment program aimed at standardizing HIV genotyping among laboratories in the Asia-Pacific region and Africa [17, 18]. FASTA files were submitted to the Stanford University HIV Drug Resistance Database [19] (Stanford HIVdb) version 6.2 for genotyping and REGA HIV-1 Subtyping Tool [20, 21] – Version 2.0 for subtyping. RAMs were defined according to the IAS-USA 2013 mutations list [10]. We excluded PI-minor mutations from our definition of RAMs as these minor variants may occur as common polymorphisms in HIV-1 non-B subtypes which is predominant in our cohort [10].

## Analyses

This study consists two analyses:

### Analysis (1): Factors associated with multi-NRTI RAMs at first-line failure

Variables adjusted in the regression model were age, sex, mode of HIV exposure, failing NRTI backbone categorized as lamivudine+stavudine, lamivudine+zidovudine and other combinations, viral load (VL) and CD4 count measured within six months prior to switch to second-line ART closest to the date of genotyping, time since ART initiation, prior AIDS-defining illnesses at time of genotyping and HIV-1 subtype. Sensitivity analysis was performed by including K65R in our definition of multi-NRTI RAMs as this mutation is associated with reduced virological response to tenofovir, didanosine, abacavir and stavudine [10].

### Analysis (2): Factors associated with VL suppression at 12 months after switch to second-line ART

We analyzed the effects of second-line treatment susceptibility on VL suppression by using the genotypic susceptibility score (GSS) for the second-line regimen, calculated based on the drug resistance scores extracted from the Stanford HIVdb. Each antiretroviral drug (ARV) was assigned a score according to the five-level Stanford HIVdb interpretation: 1.00, 0.75, 0.50, 0.25 and 0.00 for susceptible, potential low-level resistance, low-level resistance, intermediate resistance and high-level resistance, respectively [22]. The GSS was the sum of all scores for all ARVs in the patient's second-line regimen. Virological suppression was the outcome in this regression model and defined as VL <400 copies/ml at 12 months (±6 months) from switch to second-line ART. Covariates adjusted in this model were GSS, age, sex, mode of HIV exposure, VL and CD4 count at first-line failure measured within six months prior and closest to the date of switch to second-line ART, second-line NRTI backbone, time since ART initiation, previous AIDS-defining illnesses, HIV-1 subtype and second-line adherence level. As routine annual VL monitoring was performed only after cohort enrolment (time of switch to second-line ART for the majority of patients in this analysis), the regression model was adjusted by treatment duration rather than time since last virological suppression. ART adherence was recorded based on the WHO endorsed [23] self-reported 30-day Visual Analogue Scale (VAS). Adherence level was categorized based on the traditional cut-off point shown to be associated with virological failure [23]: (1) always ≥95%, (2) ever <95% and (3) no assessment (missing), within the first year of second-line regimen.

For sensitivity analyses, we replaced GSS with other resistance scores such as the sum of the actual resistance scores from the Stanford HIVdb, continuous genotypic susceptibility score (cGSS) [24], optimized background score (OBS) [25], weighted genotypic susceptibility score (wGSS) calculated according to the AntiRetroScan (version 2.0) weighting factor for drug potency (Stanford HIVdb five-level susceptibility scores multiplied by 1.2 for NNRTIs and 1.6 for boosted PIs) [26, 27], as well as a binary covariate representing whether or not the patient harboured multi-NRTI RAMs at time of first-line failure.

## Statistical methods

Logistic regression models were used in both analysis (1) and (2). In analysis (2), we attempted to categorize GSS as either above or below the optimal cut-off point calculated from the Youden Index [28]. The Youden Index cut-off point is the point on the receiver operating characteristics (ROC) curve which maximizes the difference between the true positive rate and the false positive rate. In other words, it is the cut-off point which maximizes the GSS differentiating ability for VL suppression when equal weight is given to sensitivity and specificity. We also categorized GSS around its median and compared the results to the model with the Youden Index to determine the appropriate cut-off point to be used in the final model. Regression models were fitted using backward stepwise selection process. Covariates with *p*<0.10 in the univariate model were chosen for inclusion in the multivariate model. Covariates with *p*<0.05 in the final multivariate model were considered statistically significant.

All data management and statistical analyses were performed using SAS software version 9.3 (SAS Institute Inc., Cary, NC, USA) and STATA software version 12.1 (STATA Corp., College Station, TX, USA).

## Results

Of 2023 TASER-M patients, 105 patients fit inclusion criteria and were included in the analysis. Of these, 28 patients were enrolled in the treatment-naïve group and 77 were enrolled in the treatment-experienced group. Table 1 shows there were 39/105 patients (37%) with multi-NRTI RAMs. Overall, 94% of patients were failing on a NNRTI-based regimen (61 patients on nevirapine and 38 patients on efavirenz) and 56% had prior AIDS-defining illnesses. The main NRTI backbones in the failing regimen were lamivudine+stavudine (44%) and lamivudine+zidovudine (31%). HIV-1 subtypes consist of 84% non-B and 16% B subtypes. Non-B subtypes were CRF01_AE (98%) and A1 (2%).

**Table 1:** Patient characteristics at first-line ART failure

	Total (%)	No multi-NRTI mutations (%)	Multi-NRTI mutations (%)

	*n*=105 (100)	*n*=66 (63)	*n*=39 (37)
Age at genotyping (years)			
Median (IQR)	36 (32–41)	35 (32–40)	37 (33–42)
≤30	19 (18)	13 (20)	6 (15)
31–40	59 (56)	38 (58)	21 (54)
41+	27 (26)	15 (23)	12 (31)
Sex			
Male	69 (66)	43 (65)	26 (67)
Female	36 (34)	23 (35)	13 (33)
Mode of exposure			
Heterosexual contact	76 (72)	47 (71)	29 (74)
Homosexual contact	19 (18)	15 (23)	4 (10)
Other/unknown	10 (10)	4 (6)	6 (15)
VL at genotyping (copies/ml)			
Median Log_10_ VL (IQR)	4.14 (3.65–4.94)	4.02 (3.55–4.91)	4.52 (3.95–5.00)
≤10,000	44 (42)	33 (50)	11 (28)
10,001–100,000	38 (36)	20 (30)	18 (46)
>100,000	23 (22)	13 (20)	10 (26)
CD4 at genotyping (cells/µL)			
Median (IQR)	172 (75–278.5)	206.5 (121–300.5)	83.5 (16.5–194.5)
≤200	58 (55)	30 (45)	28 (72)
>200	38 (36)	30 (45)	8 (21)
Missing	9 (9)	6 (9)	3 (8)
Years since ART initiation			
Median (IQR)	2.17 (1.07–3.91)	1.56 (0.81–3.58)	2.88 (2.09–4.29)
≤2	49 (47)	40 (61)	9 (23)
>2	56 (53)	26 (39)	30 (77)
Failing ART			
NRTI+NNRTI	99 (94)	62 (94)	37 (95)
NRTI+PI	6 (6)	4 (6)	2 (5)
Failing NRTI			
3TC+d4T	46 (44)	27 (41)	19 (49)
3TC+AZT	33 (31)	17 (26)	16 (41)
Other	26 (25)	22 (33)	4 (10)
Previous AIDS			
No	46 (44)	36 (55)	10 (26)
Yes	59 (56)	30 (45)	29 (74)
Subtype			
B	17 (16)	9 (14)	8 (21)
Non-B	88 (84)	57 (86)	31 (79)

**Figure 1:**
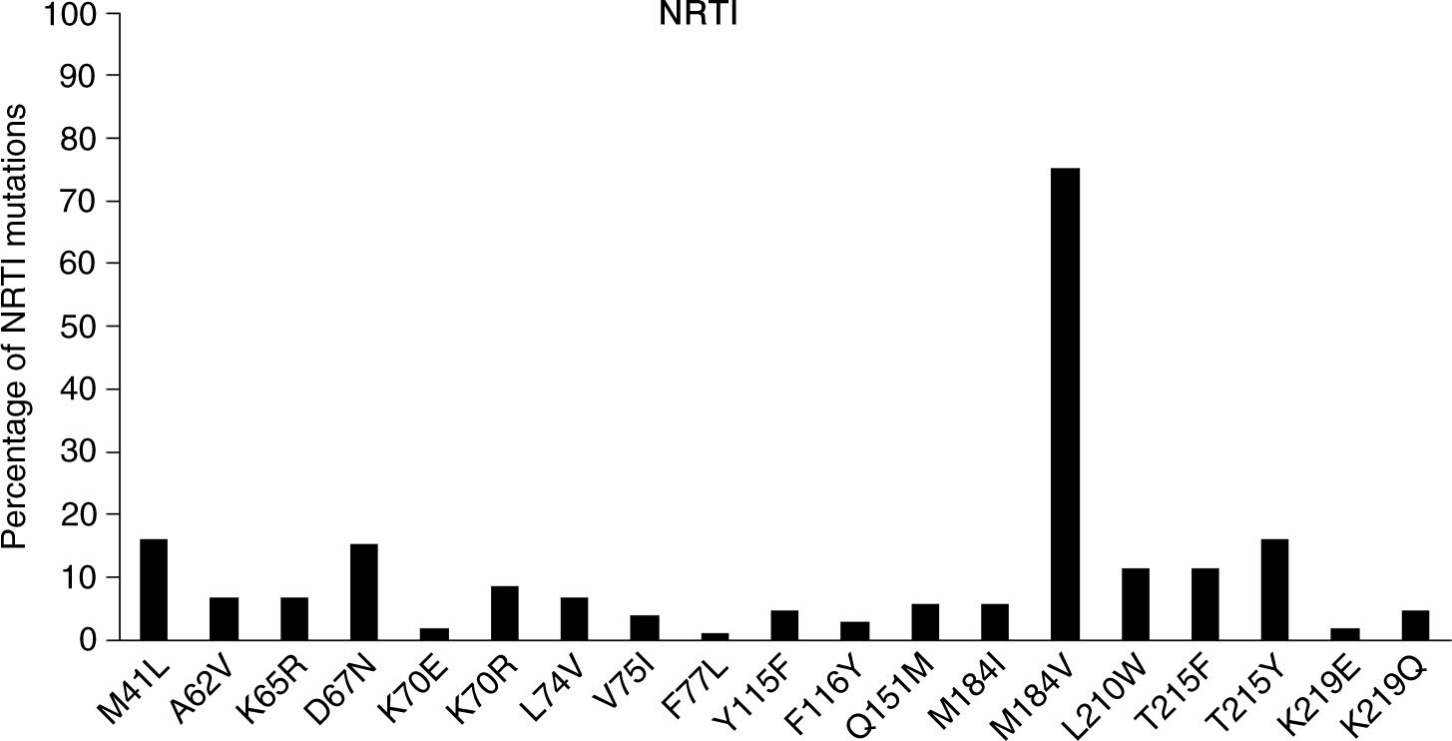
Nucleoside reverse transcriptase inhibitor (NRTI) mutations.

**Figure 2:**
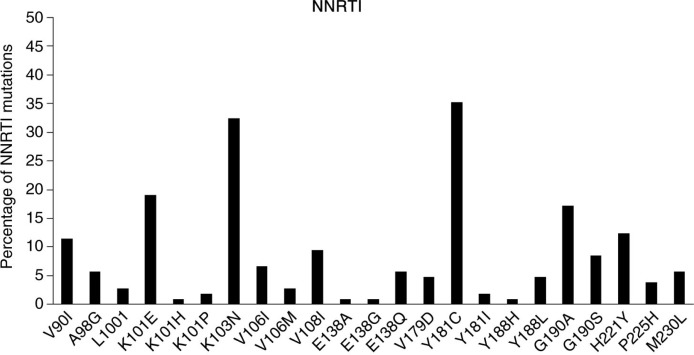
Non-nucleoside reverse transcriptase inhibitor (NNRTI) mutations.

There were 97 patients (92%) with ≥1 RAMs at first-line failure: 90 patients with NRTI RAMs, 92 with NNRTI RAMs and two with PI-major RAMs. A total of 86 patients (82%) had multi-class mutations. Figures 1 and 2 show that the most common NRTI RAM was M184V (79/105, 75%), followed by M41L and T215Y (both 17 patients each, 16%), and the most common NNRTI RAM was Y181C (37 patients, 35%), followed by K103N (34 patients, 32%). Major PI RAMs found in the two patients were M46L, G48V, V82A, N83D and L90M. Of the 39 patients harbouring multi-NRTI RAMs, there were six patients with Q151M, 24 with ≥2 TAMs and 32 with M184V+≥1 TAM. No patient harboured 69Ins. A total of 35 patients had at least 1 TAM, with the following distribution: M41L (16%), D67N (15%), K70R (9%), L210W (11%), T215Y (16%), T215F (11%), K219Q (5%) and K219E (2%).

### Analysis (1): Factors associated with multi-NRTI RAMs at first-line failure

In the univariate analysis (Table 2), variables that were significantly associated with multi-NRTI RAMs at *p*<0.10 were failing NRTI backbone (*p*=0.035), VL (*p*=0.093), CD4 count (*p*=0.009), time since ART initiation (*p*<0.001) and previous AIDS-defining illnesses (*p*=0.005). In the multivariate model, low CD4 count of ≤200 cells/µL was associated with the presence of multi-NRTI RAMs (OR=4.43, 95% CI [1.59–12.37], *p*=0.004) compared to higher CD4 count at genotyping. Patients who have been on ART for more than two years were more likely to harbour these mutations compared to those who have been treated for a shorter period of time (OR=6.25, 95% CI [2.39–16.36], *p*<0.001). All other covariates were not significant in the final multivariate model at *p*<0.05; however, VL and previous AIDS-defining illnesses were borderline significant at *p*=0.060 and *p*=0.059, respectively. When K65R was included in the sensitivity analysis, a total of 46/105 (44%) patients were classified as harbouring multi-NRTI RAMs. Variables remaining significant in the final multivariate model were CD4 ≤200 cells/µL (OR= 5.28, 95% CI [2.01–13.87], *p*=0.001) compared to CD4 >200 cells/µL, and time on ART >2 years (OR=3.45, 95% CI [1.42–8.36], *p*=0.006), compared to ≤2 years. As the results of this sensitivity analysis are similar to those presented in Table 2, the absence of K65R in our multi-NRTI RAM definition did not significantly alter the analysis outcome.

**Table 2:** Factors associated with multi-NRTI RAMs at first-line ART failure

			Univariate				Multivariate			
	
	Total patients	Multi-NRTI RAMs	OR	95% CI	*p*	Global p*	OR	95% CI	*p*	Global p*
Age at genotyping (years)										
≤30	19	6	1			0.630	1			0.268
31–40	59	21	1.20	0.40–3.61	0.749		0.49	0.12–1.91	0.302	
41+	27	12	1.73	0.51–5.93	0.381		1.13	0.27–4.71	0.865	
Sex										
Male	69	26	1			0.874	1			0.972
Female	36	13	0.93	0.41–2.16	0.874		0.98	0.38–2.54	0.972	
Mode of exposure										
Heterosexual contact	76	29	1			0.128	1			0.788
Homosexual contact	19	4	0.43	0.13–1.43	0.169		0.77	0.20–2.92	0.700	
Other/unknown	10	6	2.43	0.63–9.35	0.196		1.51	0.33–6.90	0.598	
Failing NRTI backbone										
3TC+d4T	46	19	1			0.035	1			0.172
3TC+AZT	33	16	1.34	0.54–3.29	0.527		1.74	0.62–4.89	0.293	
Other	26	4	0.26	0.08–0.87	0.029		0.46	0.12–1.80	0.262	
VL at genotyping (copies/ml)										
≤10,000	44	11	1			0.093	1			0.060
10,001–100,000	38	18	2.70	1.06–6.87	0.037		3.73	1.24–11.23	0.019	
>100,000	23	10	2.31	0.79–6.73	0.126		2.53	0.69–9.20	0.160	
CD4 at genotyping (cells/µL)										
≤200	58	28	3.50	1.37–8.91	0.009		**4.43**	**1.59**–**12.37**	**0.004**	**0.004**
>200	38	8	1			0.009	1			
Missing	9	3	1.87	0.38–9.20	0.438		1.59	0.30–8.58	0.587	
Years since ART initiation										
≤2	49	9	1			<0.001	1			**<0.001**
>2	56	30	5.13	2.10–12.53	<0.001		**6.25**	**2.39**–**16.36**	**<0.001**	
Previous AIDS										
No	46	10	1			0.005	1			0.059
Yes	59	29	3.48	1.46–8.28	0.005		2.52	0.97–6.56	0.059	
Subtype										
B	17	8	1			0.358	1			0.328
Non-B	88	31	0.61	0.21–1.74	0.358		0.54	0.16–1.85	0.328	

* Global *p*-values are test for heterogeneity excluding not tested or missing values.

Results in bold represent significant covariates in the final model. Non-significant factors were presented in the multivariate model adjusted for significant predictors.

3TC=lamivudine; d4T=stavudine; AZT=zidovudine.

### Analysis (2): Factors associated with VL suppression at 12 months after switch to second-line ART

Out of 105 patients, 87 had VL testing at 12 months after switching to second-line therapy. The proportion of patients who achieved VL suppression was 74/87 (85%). Second-line regimen consisted mainly of NRTI+PI (98%). The NRTI backbones were lamivudine+tenofovir (32%), zidovudine+tenofovir (16%) and other combinations (52%). The proportions of individual NRTI combinations included in the other category were <10% each. The second-line PI component consisted of boosted lopinavir (86%), atazanavir (7%), indinavir (6%) and darunavir (1%).

The GSS was calculated from the Stanford HIVdb resistance interpretations for the second-line regimen. GSS had a range of 1.00–4.00, with a median of 2.00 and IQR (1.50–3.00). The GSS cut-off point obtained from the Youden Index was 1.25 (Table 3). Using the Youden Index, the total number of patients with GSS <1.25 was 3/87, with two patients achieving VL suppression. The 95% CI from the univariate analysis was (0.25–35.72). When using the median GSS as the cut-off point, there were 24/87 patients having GSS <2.00, with 20/24 VL suppression. The 95% CI using this median cut-off was (0.33–4.34). Since categorizing GSS around its median provided a more evenly distributed data with a much smaller 95% CI, we chose the median, rather than the Youden Index, as the cut-off point for the GSS covariate in the regression model.

**Table 3:** Sensitivity, specificity and Youden Index for a range of genotypic susceptibility score (GSS)

GSS	Sensitivity	Specificity	Sensitivity-(1-Specificity)	Youden Index
1	1.00	0.00	0.00	
**1.25**	**0.97**	**0.08**	**0.05**	**0.05**
1.5	0.82	0.15	−0.02	
1.75	0.74	0.23	−0.03	
2	0.73	0.31	0.04	
2.25	0.47	0.46	−0.07	
2.5	0.41	0.46	−0.13	
2.75	0.38	0.54	−0.08	
3	0.27	0.54	−0.19	
4	0.01	1.00	0.01	

Note: Bold values represent the GSS, sensitivity, and specificity associated with the Youden Index.

In both the univariate and multivariate analyses (Table 4), the only variable that was significantly associated with VL suppression was ART adherence. By always having adherence level ≥95% from the self-reported VAS in the first year of second-line regimen, the odds for achieving VL suppression was at least nine times higher than those who ever reported <95% during the year (OR=9.33, 95% CI (2.43–35.81), *p*=0.001). The GSS variable was not significantly associated with VL suppression in both univariate and multivariate models.

**Table 4:** Factors associated with virological suppression at 12 months after switch to second-line ART

			Univariate				Multivariate			
	
	Total patients	VL suppression	OR	95% CI	*p*	Global p*	OR	95% CI	*p*	Global p*
GSS										
<2.00	24	20	1			0.781	1			0.544
≥2.00	63	54	1.20	0.33–4.34	0.781		1.56	0.37–6.56	0.544	
Age at switch to second-line ART (years)										
≤36	44	36	1			0.395	1			0.313
>36	43	38	1.69	0.51–5.65	0.395		1.98	0.52–7.46	0.313	
Sex										
Male	58	48	1			0.400	1			0.765
Female	29	26	1.81	0.46–7.15	0.400		1.26	0.28–5.59	0.765	
Mode of exposure										
Heterosexual contact	60	52	1			0.386	1			0.646
Homosexual contact	17	15	1.15	0.22–6.02	0.865		1.22	0.21–7.20	0.823	
Other/Unknown	10	7	0.36	0.08–1.68	0.193		0.47	0.08–2.71	0.402	
VL at switch to second-line ART (copies/ml)										
≤10,000	36	32	1			0.698	1			0.946
10,001–100,000	33	27	0.56	0.14–2.20	0.409		0.82	0.18–3.77	0.800	
>100,000	18	15	0.63	0.12–3.15	0.569		1.06	0.17–6.43	0.953	
CD4 at switch to second-line ART (cells/µL)										
≤200	50	41	1			0.545	1			0.167
>200	31	27	1.48	0.41–5.30	0.545		2.89	0.64–13.02	0.167	
Missing	6	6	N/A				N/A			
Second-line NRTI backbone										
3TC+TDF	28	23	1			0.565	1			0.234
AZT+TDF	14	11	0.80	0.16–3.95	0.781		0.85	0.14–5.14	0.862	
Other	45	40	1.74	0.45–6.65	0.419		3.45	0.67–17.74	0.138	
Years from ART initiation to second-line ART										
≤2	40	36	1			0.240	1			0.067
>2	47	38	0.47	0.13–1.66	0.240		0.23	0.05–1.11	0.067	
Previous AIDS at switch to second-line ART										
No	39	34	1			0.618	1			0.603
Yes	48	40	0.74	0.22–2.46	0.618		0.71	0.19–2.62	0.603	
Subtype										
B	12	9	1			0.301	1			0.274
Non-B	75	65	2.17	0.50–9.39	0.301		2.52	0.48–13.13	0.274	
Adherence in the first 12 months of second-line ART										
Ever <95%	16	9	1			0.001	1			**0.001**
Always ≥95%	65	60	9.33	2.43–35.81	0.001		**9.33**	**2.43**–**35.81**	**0.001**	
Missing	6	5	3.89	0.37–41.32	0.260		3.89	0.37–41.32	0.260	

* Global *p*-values are test for heterogeneity excluding not tested or missing values.

Results in bold represent significant covariates in the final model. Non-significant factors were presented in the multivariate model adjusted for significant predictors.

3TC=lamivudine; TDF=tenofovir; AZT=zidovudine; VL=viral load.

In the sensitivity analyses, we first replaced the GSS with the sum of the actual resistance scores for each ARV obtained directly from the Stanford HIVdb. The resistance scores ranged from −20 to 200, with a median of 45 and IQR (0–75). The resistance scores were not significantly associated with VL suppression (*p*=0.868). Further analyses were also performed using cGSS, OBS, and multi-NRTI RAMs binary covariate. These variables were also not associated with virological outcome (*p*=0.664, *p*=0.395 and *p*=0.744, respectively). The wGSS had a median of 2.6 with IQR (2.10–3.60). There was no difference between wGSS scores above and below the median in their association with VL suppression (OR=1.12, 95% CI [0.31–4.04], *p*=0.861).

## Discussion

A high proportion of patients in this sub-analysis of TASER-M who failed first-line ART harboured drug resistance mutations (92%). Multi-NRTI RAMs were found in 37% of the patients. Six patients had Q151M, 24 had ≥2 TAMs and 32 had M184V+≥1 TAM. Low CD4 count and longer duration on ART were linked to the presence of multi-NRTI RAMs at first-line failure. However, these mutations and the resulting treatment susceptibility did not have a significant effect on the virological response of second-line therapy. Adherence level was the only significant factor associated with achieving VL suppression 12 months after switching to second-line ART.

A high prevalence of drug resistance mutations is commonly seen in patients failing first-line ART. Our findings were consistent with previous reports which showed that NNRTI RAMS were the most common, followed by NRTI and PI RAMs [6, 29, 30]. Our study also found that approximately one-third of the patients harboured multi-NRTI RAMs. This prevalence was higher than the 12.7% reported from a Cambodian study [31], although it is important to note that multi-NRTI RAMs in that study included Q151M, K65R and 69Ins.

Results from analysis (1), which investigated factors associated with multi-NRTI RAMs at first-line failure, indicate that the two significant factors were low CD4 count and longer time on ART. Although our study found that VL was only marginally significant at *p*=0.060, it is known that viraemia can be associated with the accumulation of drug resistance mutations, in particular, NRTI RAMs. A study in Africa has found that NRTI cross-resistance was associated with higher VL in the univariate analysis and was borderline significant (*p*=0.052) in the adjusted model [32]. Another African study also reported a 76% increase in NRTI RAMs during prolonged periods of viraemia [33]. VL monitoring in resource-limited settings is recommended every 12 months after ART initiation to detect treatment failure if VL testing is routinely available [15, 34]. Therefore, it is possible that patients enrolled as treatment-experienced in our cohort may have had periods of viraemia prior to entering TASER-M, and therefore may be at higher risk of drug resistance accumulation.

The association of low CD4 level with RAMs was consistent with a Chinese study [35] which also found that CD4 count of <200 cells/µL was associated with the development of drug resistance at ART failure. The length of exposure to ART has also played a significant role. Our study found that having been exposed to ART for more than two years greatly increases the chance of having multi-NRTI RAMs. Other studies also confirmed this association [32, 35–37]; however, we hypothesize that adherence to first-line regimen may also act as a confounder for resistance. We were unable to adjust for this confounding effect due to the unavailability of adherence data prior to enrolment to TASER-M. Our study also did not find any significant difference between the use of lamivudine+stavudine, lamivudine+zidovudine and other NRTI combinations in the association with the development of multi-NRTI RAMs.

The second-line virological outcome analysis (analysis [2]) showed the proportion of patients achieving virological suppression was high (85%) with majority of patients receiving PI-based regimen. Comparable rates were also seen in rural South Africa where 72% achieved VL <400 copies/ml at 12 months and VL <50 copies/ml were seen in 61% of patients [38]. It was also shown in our study that by always maintaining adherence levels at ≥95% in the first year of second-line regimen, VL suppression was more likely to be achieved. ARV susceptibility, either in the form of GSS, resistance scores, cGSS, OBS or wGSS, as well as the presence of multi-NRTI RAMs, proved not to have a significant impact on virological outcome. This may reflect the low cross-resistance between NNRTI- and PI-based regimens and high genetic barrier of PIs, the small sample size of the study or the relatively high susceptibility scores in our patients. However, the findings were in parallel with previous studies [39–41] which confirmed that adherence level was the main factor in determining treatment outcome of second-line therapy.

The Department of Health and Human Services Antiretroviral Guidelines for Adults and Adolescents (February 2013) recommend that after first-line failure has occurred, the new ARV regimen should contain at least two fully active drugs [42]. The susceptibility score for a regimen with at least two fully active ARVs, that is “susceptible” Stanford HIVdb predictions, would equate to GSS ≥2. The median GSS for our patient group was 2.00 with 63/87 patients having GSS of two or more. This suggests that 72% of our patients had a high degree of susceptibility to second-line ART. As the majority of patients switched to a PI-based regimen with only two harbouring PI-major RAMs, it appears that constructing a robust second-line regimen is a feasible goal for most patients. Of three patients with a GSS=1, one patient (33%) had virological failure.

Among the TASER-M sites, selection of the second-line regimen after first-line ART failure varied across different clinical settings. Sites with relatively quick sequencing turn-around time were able to select drugs and start patients on second-line regimen after the genotypic resistance profile had been completed. However, at some sites, patients were switched to second-line ART prior to completion of sequence amplification, either due to the long sequencing time, or limited options available for second-line ART. The WHO guidelines recommend selection of second-line ARVs based on the combination of ARVs received during first-line therapy, rather than the presence of drug resistance results showing mutations at failure [15]. Because of the relatively small sample size of our patient group and since more than half of our patients were switched with resistance test results available, our analyses should not be interpreted as either evidence for or against these WHO guidelines.

The limitations of the study were the limited sample sizes of patients who failed first-line ART and those with VL available at 12 months after switching to second-line. We were also unable to adjust for site or country differences due to relatively few data points in some of the categories. Adherence to first-line ART was not available for the majority of patients. This is because more than 70% of patients included in the analysis were enrolled in TASER-M in the treatment-experienced group. For this group, prospective adherence data was collected at time of switch to second-line onwards. Since no retrospective self-reported adherence was available, it was not possible to determine the confounding effects of first-line ART adherence on the presence of multi-NRTI RAMs.

## Conclusions

TASER-M patients failing first-line ART reported high prevalence of multi-NRTI RAMs with many switching from a NNRTI-based regimen to a PI-based regimen. Although multi-NRTI RAMs can compromise treatment choices of second-line therapy, it has been shown that virological suppression can be achieved if high level of adherence was maintained. ART adherence proved to be a significant contributor to the success of second-line therapy in this patient group who mostly switched to a highly susceptible treatment regimen. This emphasizes the importance of appropriate adherence counselling and intervention which should be continued well beyond first-line therapy. Further studies with larger sample sizes and longer follow-up time are required to determine if high ART adherence level can maintain its positive effects over time in the presence of drug resistance mutations during second-line therapy.

## Genbank accession numbers

The Genbank accession numbers obtained from the sequences used in this study are KJ868849-KJ869058. The accession numbers categorized according to the failing first-line NRTI+NNRTI combination are as follows: **3TC+d4T+NVP:**KJ868870,KJ868873,KJ868881,KJ868888,KJ868889,KJ868890,KJ868891,KJ868892,KJ868894,KJ868901,KJ868902,KJ868908,KJ868909,KJ868912,KJ868914,KJ868915,KJ868917,KJ868919,KJ868921,KJ868922,KJ868927,KJ868928,KJ868929,KJ868932,KJ868933,KJ868934,KJ868935,KJ868940,KJ868947,KJ868950,KJ868951,KJ868975,KJ868978,KJ868986,KJ868993,KJ868994,KJ868995,KJ868996,KJ868997,KJ868999,KJ869006,KJ869007,KJ869013,KJ869014,KJ869017,KJ869019,KJ869020,KJ869022,KJ869024,KJ869026,KJ869027,KJ869032,KJ869033,KJ869034,KJ869037,KJ869038,KJ869039,KJ869040,KJ869045,KJ869052,KJ869055,KJ869056;**3TC+AZT+NVP:**KJ868850,KJ868864,KJ868871,KJ868882,KJ868883,KJ868885,KJ868898,KJ868900,KJ868904,KJ868905,KJ868916,KJ868918,KJ868920,KJ868930,KJ868938,KJ868939,KJ868943,KJ868945,KJ868946,KJ868948,KJ868949,KJ868952,KJ868955,KJ868969,KJ868976,KJ868987,KJ868988,KJ868990,KJ869003,KJ869005,KJ869009,KJ869010,KJ869021,KJ869023,KJ869025,KJ869035,KJ869043,KJ869044,KJ869048,KJ869050,KJ869051,KJ869053,KJ869054,KJ869057;**3TC+d4T+EFV:**KJ868849,KJ868857,KJ868862,KJ868868,KJ868899,KJ868907,KJ868910,KJ868911,KJ868925,KJ868936,KJ868937,KJ868942,KJ868954,KJ868962,KJ868967,KJ868973,KJ869004,KJ869012,KJ869015,KJ869016,KJ869030,KJ869041,KJ869042,KJ869047;**3TC+AZT+EFV:**KJ868851,KJ868865,KJ868866,KJ868887,KJ868893,KJ868896,KJ868897,KJ868924,KJ868944,KJ868956,KJ868970,KJ868971,KJ868992,KJ868998,KJ869001,KJ869002,KJ869029,KJ869049;**3TC+TDF+EFV:**KJ868875,KJ868878,KJ868879,KJ868880,KJ868884,KJ868886,KJ868913,KJ868980,KJ868983,KJ868984,KJ868985,KJ868989,KJ868991,KJ869018;**3TC+ABC+EFV:**KJ868853,KJ868854,KJ868856,KJ868859,KJ868860,KJ868861,KJ868958,KJ868959,KJ868961,KJ868964,KJ868965,KJ868966;**3TC+TDF+NVP:**KJ868872,KJ868874,KJ868895,KJ868923,KJ868931,KJ868977,KJ868979,KJ869000,KJ869028,KJ869036;**3TC+d4T:**KJ868867,KJ868926,KJ868941,KJ868972,KJ869031,KJ869046;**FTC+TDF+EFV:**KJ868876,KJ868877,KJ868906,KJ868981,KJ868982,KJ869011;**3TC+AZT:**KJ868852,KJ868863,KJ868957,KJ868968;**3TC+ABC+NVP:**KJ868855,KJ868960;**3TC+AZT+TDF+NVP:**KJ868903,KJ869008;**3TC+DDI+EFV:**KJ868858,KJ868963;**AZT+DDI+NVP:**KJ868869,KJ868974;**FTC+TDF:**KJ868953,KJ869058.
